# Expression of miR-296-5p as predictive marker for radiotherapy resistance in early-stage laryngeal carcinoma

**DOI:** 10.1186/s12967-015-0621-y

**Published:** 2015-08-12

**Authors:** Danielle Maia, Ana Carolina de Carvalho, Maria Aderuza Horst, André Lopes Carvalho, Cristovam Scapulatempo-Neto, Andre Luiz Vettore

**Affiliations:** Laboratory of Molecular Cancer Biology, Department of Biological Sciences, Federal University of São Paulo, Rua Pedro de Toledo, 669-11° andar, São Paulo, SP 04039-032 Brazil; Molecular Oncology Research Center, Barretos Cancer Hospital, Rua Antenor Duarte Vilela, 1331, Barretos, SP 14784-400 Brazil; Department of Head and Neck Surgery, Barretos Cancer Hospital, Rua Antenor Duarte Villela, 1331, Barretos, 14784-400 Brazil; Department of Pathology, Barretos Cancer Hospital, Rua Antenor Duarte Villela, 1331, Barretos, 14784-400 Brazil; Cancer and Stem Cell Biology Program, Duke-NUS Graduate Medical School, Singapore, Singapore

**Keywords:** Early-stage laryngeal cancers, Molecular marker, MicroRNAs, Radioresistance, miR-296-5p

## Abstract

**Purpose:**

Definitive radiation therapy is the mainstay of treatment for early stage laryngeal squamous cell carcinoma (LSCC). However, up to 30% of the patients do not respond to radiotherapy. Unfortunately, we are unable to predict which tumors are likely to respond to radiation, and which will be resistant and persist. Therefore, the development of novel markers to predict response to radiotherapy is urgently needed. This study was designed to evaluate the expression pattern of microRNAs (miRNAs) in LSCC in order to identify markers capable of segregating radioresistant and radiosensitive tumors and to investigate the relationship between the expression of these miRNAs and the prognosis of LSCC.

**Methods:**

The expression profile of 667 miRNAs was determined in an initial screening of nine early-stage LSCC samples (5 radioresistant and 4 radiosensitive) using TaqMan Low-Density Array (TLDA). Real-time polymerase chain reactions were performed to validate the expression of selected miRNAs in an expanded LSCC cohort (20 radioresistant and 14 radiosensitive). The miRNA expression level was scored as high or low based on the median of the expression in the LSCC samples.

**Results:**

A comprehensive miRNA expression profiling enabled the identification of four miRNAs (miR-296-5p miR-452, miR-183* and miR-200c) differentially expressed in radioresistant LSCC. Moreover, the analysis of additional 34 LSCC samples, confirmed the expression of miR-296-5p as significantly related to radioresistance (p = 0.002) as well as an association of this marker with recurrence (p = 0.025) in early stage laryngeal cancer.

**Conclusions:**

This study indicates that miR-296-5p expression is associated with resistance to radiotherapy and tumor recurrence in early stage LSCC, showing the feasibility of this marker as a novel prognostic factor for this malignance. Furthermore, miR-296-5p expression could be helpful in the identification of tumors resistant to radiotherapy; thus aiding the clinicians in the choice of the best therapeutic scheme to be used in each case.

**Electronic supplementary material:**

The online version of this article (doi:10.1186/s12967-015-0621-y) contains supplementary material, which is available to authorized users.

## Background

Laryngeal cancer represents the second most common site of head and neck tumors [1, 2], and has squamous cell carcinoma as the most important histology [2]. Accounting for approximately 160,000 new cases per year and for 2.5% of all tumors in males, LSCC remains a major disease burden worldwide. Treatment options comprise surgery, radiotherapy, chemotherapy or a combination of modalities [3, 4]. Despite refinement of multimodal therapies, the 5-year relative survival percentage has been stable at around 60 for the last decades for early laryngeal cancer [5].

Definitive radiation therapy is the mainstay of treatment for early stage (T1-T2 N0) laryngeal squamous cell carcinoma (LSCC). However, 6–15% of patients with stage 1 disease and 20–31% of patients with stage 2 disease do not respond to radiotherapy [6]. If a patient fails radiotherapy, surgery is the main treatment option that may offer a cure, and up to 50% will require a total laryngectomy as salvage treatment. Moreover, when used as salvage surgery after a failed radiotherapy course, laryngectomy presents an increased complication rate [7]. Remarkably, for these failed cases, definitive cancer cure is delayed by the course of radiotherapy with a risk of tumor progression, adversely affecting patient prognosis still further. Unfortunately, we are unable to predict which tumors are likely to respond to radiation, and those, which will be resistant and persist. Therefore, it would be desirable to find novel and valuable markers to predict beforehand which patients will benefit from radiotherapy.

Relevant clinical factors associated with local recurrence after radiotherapy are tumor size, tumor stage, overall treatment time and radiotherapy fraction size [8–10]. Treatment choice is now mainly based on T-stage [11], however, this is insufficient to warrant recurrence-free survival as observed in heterogeneous responses and survival rates presented by LSCC patients [6].

Molecular markers have the potential to help in the selection of treatment through response prediction and determination of cancer prognosis [12, 13] Previous studies have identified few molecular markers, such as p53, cyclin D1, EGFR, VEGF, IGF1R, COX2 and Ki67, associated with local relapse and overall survival in early laryngeal carcinoma [12, 14–16]. However, some of these findings are conflicting and none of these markers are being used in clinical practice.

MicroRNAs (miRNAs) are non-coding RNAs with 18–25 nucleotides, described as negative regulators of gene expression in a variety of multicellular organisms. These small molecules harbor the capacity to bind and silence specific messenger RNA (mRNA) targets, inducing their degradation, or inhibiting their translation [17]. MicroRNAs play important roles in various biological processes, such as apoptosis, cell proliferation, cell differentiation, tumorigenesis, and multidrug resistance (MDR) [18]. Emerging evidences revealed that miRNA expression patterns are significantly changed in cancer cells submitted to ionizing radiation [19]. Many studies have shown modulation of radiosensitivity by altering miRNA levels in various malignances, such as lung cancer cells in vitro [20] and breast cancer cells in vivo [21].

The purposes of this study were to assess the global miRNA expression profile of patients with T1–T2 N0 laryngeal cancer treated with primary radiation therapy, in order to identify miRNAs able to segregate radioresistant and radiosensitive tumors, thus serving as markers to predict radiotherapy resistance. Moreover, the role of miRNAs in prognosis will be assessed by searching for associations between the expression of selected miRNAs and clinical and pathological parameters of LSCC patients.

## Methods

### Patients and tissue samples

This retrospective study included 34 patients with primary LSCC tumors (glottic and supraglottic) treated with radiotherapy as first and single modality treatment with curative intent at the Barretos Cancer Hospital, Barretos, São Paulo, Brazil, between 2000 and 2010. Diagnosis of LSCC was determined according to the WHO criteria.

Patients were identified as harboring radioresistant or radiosensitive tumors depending upon their response to radiotherapy. Tumors were staged according to the TNM classification [22] with all cases being clinically T1–T2, clinically nodal negative (N0) and negative for distant metastases (M0) at the time of treatment.

The radioresistant cohort consisted of 20 LSCC patients. The criteria for radioresistant tumors were: (a) radiotherapy had to be given as a single modality treatment with curative intent for a biopsy-proven squamous cell carcinoma of the larynx; (b) biopsy-proven recurrent squamous cell carcinoma, with the recurrence occurring at the local or locoregional original anatomical site after finishing the course of radiotherapy.

The radiosensitive cohort of tumors was comprised of 14 LSCC patients. The criteria for radiosensitive tumors were: (a) radiotherapy had to be given as a single modality treatment with curative intent for a biopsy-proven squamous cell carcinoma of the larynx; (b) post treatment, patients had a minimum follow-up of 3 years with no evidence of a recurrent laryngeal tumor. The definition for LSCC recurrence adopted in this study is the presence of tumor cell in the same site of the primary tumor or radiological evidence of locoregional disease after finishing the course of radiotherapy and during the first 3 years of follow-up.

This study was approved by the local IRB for the use of archival biopsy material and patient clinical data collection. Medical records of patients were reviewed for standard demographic data, pretreatment classification, tumor staging, radiation therapy parameters, and disease outcome.

### Radiotherapy

Radiation treatment parameters were as follows: bilateral laryngeal opposed wedged fields using beam energies of 6 MV, 5 fractions per week, without planned treatment breaks. Patients were treated with daily fractions of 2–2.25 Gy to a total median dose of 66 Gy (range 49.5–79 Gy) over 47 days (range 38–79 days).

### RNA isolation and cDNA synthesis

Biopsies sections (5-μm thick) were performed from pre-treatment archival tissue blocks of the radioresistant and radiosensitive tumors. Total RNA was isolated using the miRNeasy FFPE Kit (Qiagen, Hilden, Germany), according to the manufacturer’s instructions. Ten nanograms of total RNA from each sample were subjected to reverse transcription using the TaqMan MicroRNA Reverse Transcription Kit (Applied Biosystems, Foster City, CA, USA) and specific stem-loop primers for each of the microRNAs selected, in accordance with the manufacturer’s recommendations.

### Global microRNA profiling

Global microRNA expression profiling of FFPE samples in a discovering set comprised of 9 patients (5 from the radioresistant group and 4 from the radiosensitive group) was performed using the TaqMan Human MicroRNA Cards Set v2.0 (Applied Biosystems), allowing the evaluation of the expression level of 667 human microRNAs. Forty nanograms of total RNA from each sample was reverse transcribed into cDNA using the TaqMan miRNA Reverse Transcription Kit (Applied Biosystems) and Megaplex RT Primers (Applied Biosystems). Next, the product obtained from the RT reactions was pre-amplified using the TaqMan PreAmp Master Mix Kit (Applied Biosystems) and Megaplex PreAmp Primers (Applied Biosystems). The amplified-cDNA was then transferred to the TaqMan Human MicroRNA Cards Set v2.0 and the amplification was carried out in the 7900HT Real-Time PCR System (Applied Biosystems). The data obtained was analyzed using the software DataAssist v3.0 (Applied Biosystems). The fold-change difference between radioresistant and radiosensitive cases was calculated using the 2^−ΔΔCt^ method [23]. The *small nuclear RNA U6* was used as endogenous control and the radiosensitive cases were assigned as reference. Cases were scored as differentially expressed if a 4-fold-change increase was observed.

### Validation of the differentially expressed microRNAs

The expression level of the miRNAs selected for the validation step was evaluated in a total of 34 samples (20 radioresistant and 14 radiosensitive) using individual TaqMan MicroRNA Assays (Applied Biosystems). Each assay was conducted using the TaqMan MicroRNA Reverse Transcription kit (Applied Biosystems) according to the manufacturer’s protocols. Briefly, 10 ng of total RNA were reverse-transcribed using MultiScribe Reverse Transcriptase (Applied Biosystems) and a stem-loop primer (Applied Biosystems). The mixture was incubated at 16°C for 30 min, 42°C for 30 min and 85°C for 5 min. Quantitative RT-PCR (qRT-PCR) was performed using TaqMan PCR kit (Applied Biosystems) on a 7500 Fast Real-Time PCR System (Applied Biosystems). Three technical replicates of each sample were performed for every microRNA. To evaluate the differential expression of each microRNA between radioresistant and radiosensitive cases, the 2^−ΔΔCt^ method was employed [23]. Mean Ct values of *U6 small nuclear RNA* was used for normalization.

### Statistical analysis

To search for differentially expressed microRNAs between both groups in the global miRNA expression profiling, ΔCt values from each microRNA were evaluated using the t-Student test with the Benjamini–Hochberg adjustment for false discovery rate (FDR) as implemented in the DataAssist software v3.0 (Applied Biosystems). The individual assay results were analyzed after normalization of data. ΔCT values of microRNAs assayed in the validation step using individual TaqMan assays were used for comparisons between groups using Mann–Whitney U test for non-normal distribution.

The miRNA levels measured during the validation step were converted into discrete variables by splitting the samples into two classes (high and low expression) using the ΔCt median level considering all samples evaluated as cutoff. The Chi square test and Fisher’s exact test were used to evaluate the associations between miRNA expression and clinical variables, as appropriate. The Kaplan–Meier method was used to estimate disease-free survival (DFS) of patients, and the log-rank test was used to examine the differences between groups. The DFS was defined as the time interval between the date of the end of the radiation therapy and the date of diagnosis of the first recurrence, or last date of follow-up if recurrence was not observed. A p value of <0.05 indicated the presence of statistically significant difference between groups. All statistical analyses were performed using SPSS statistics 20.0.

## Results

### Characteristics of the patients

The clinical and histological characteristics of the 34 patients enrolled in this study are listed in Table 1. Patients were mainly male (88.2%), with age ranging from 39 to 85 years (median 62.5 years). Tobacco use (current or former) was reported by 91.2% of the patients. Primary tumor sites were predominantly glottic (79.4%) and tumor stage at diagnosis was T1 in 47.1% and T2 in 52.9% of the cases. Most patients (70.6%) received at least 70 Gy in a range from 6,300 to 7,020 cGy (total median dose, 66 Gy), within a median delivery interval of treatment of 55 days (range 38–79 days). None of the clinical and therapeutic variables were correlated with the radioresistant tumors, as noted in Table 1.

**Table 1:** Clinical and pathological data of the patients enrolled in the study

	Number of cases (%)	Radiosensitive	Radioresistant	X^2^ (p value)
		n (%)	n (%)	
Gender				
Male	30 (88.2)	12 (40.0)	18 (60.0)	1.0
Female	4 (11.8)	2 (50.0)	2 (50.0)	
Age				
≤60 years	16 (47.1)	5 (31.2)	11 (68.8)	0.51
>60 years	18 (52.9)	9 (50.0)	9 (50.0)	
Tobacco consumption				
Yes	31 (91.2)	14 (45.2)	17 (54.8)	0.25
No	3 (8.8)	0 (0)	3 (100.0)	
Tumor site				
Supraglottic	7 (20.6)	2 (28.6)	5 (71.4)	1.0
Glottic	27 (79.4)	12 (44.4)	15 (55.6)	
Tumor stage				
I	16 (47.1)	8 (50)	8 (50)	0.49
II	18 (52.9)	6 (33.3)	12 (66.7)	
Radiotherapy dose				
<70 Gy	10 (29.4)	5 (50.0)	5 (50.0)	0.7
≥70 Gy	24 (70.6)	9 (37.5)	15 (62.5)	
Treatment time				
≤55 days	20 (58.8)	9 (45.0)	11 (55.0)	0.72
>55 days	14 (41.2)	5 (37.5)	9 (64.3)	

### MicroRNA expression in LSCC samples

Comprehensive miRNA profiles were generated from LSCC samples collected from radioresistant (n = 5) and radiosensitive (n = 4) patients using a quantitative RT-PCR array platform (Additional file 1: Table S1). miRNAs were considered differentially expressed in concordance with two criteria: (1) miRNAs with FDR-adjusted p value <0.3, as previously suggested [24], and (2) an increase in the expression level of the radioresistant group in comparison to the radiosensitive group greater than 4-fold. Thus, by using a FDR-adjusted p value <0.3, 42 miRNAs could be considered as differentially expressed between radioresistant and radiosensitive samples considering a 4-fold difference. From those, based in the expression level and literature searching, 15 miRNA were selected for further analysis. All miRNAs that were at least 2-fold up-regulated or down-regulated are presented in Table 2.

**Table 2:** A list of miRNAs differentially expressed according to the TaqMan Human MicroRNA Array

Up-regulated			Down-regulated		
microRNA	Fold-change	FDR-adjusted p-value	microRNA	Fold-change	FDR-adjusted p value
hsa-miR-642	2.02	0.3869	hsa-miR-106b	2.01	0.2162
hsa-miR-744	2.02	0.4254	hsa-miR-302b	2.05	0.5657
hsa-miR-221	2.03	0.3475	hsa-miR-136*	2.06	0.5306
hsa-miR-193b*	2.07	0.3359	hsa-miR-127-5p	2.09	0.5626
hsa-miR-193b	2.08	0.4149	hsa-miR-152	2.09	0.3678
hsa-miR-18b	2.11	0.3012	hsa-miR-34c-5p	2.09	0.4936
hsa-miR-222*-	2.15	0.4874	hsa-miR-199a-5p	2.11	0.6033
hsa-miR-944	2.16	0.4246	hsa-miR-190b	2.12	0.6416
hsa-miR-766-	2.21	0.3723	hsa-miR-140-3p	2.14	0.4081
hsa-miR-106b*	2.22	0.3155	hsa-let-7i*	2.16	0.5682
hsa-miR-942	2.35	0.3275	hsa-miR-195	2.17	0.508
hsa-miR-135a	2.41	0.1506	hsa-miR-302c	2.17	0.5853
hsa-miR-652	2.41	0.2572	hsa-miR-488	2.17	0.5469
hsa-miR-124	2.42	0.3872	hsa-miR-411*	2.17	0.5271
hsa-miR-105	2.51	0.2277	hsa-miR-21	2.18	0.3333
hsa-miR-923	2.62	0.5362	hsa-miR-590-5p	2.18	0.37
hsa-miR-138	2.71	0.2162	hsa-miR-29c	2.23	0.4804
hsa-miR-181a-2*	2.78	0.2034	hsa-miR-454	2.24	0.3152
hsa-miR-23b	2.84	0.2235	hsa-miR-10b*	2.25	0.5091
hsa-miR-224	2.85	0.2626	hsa-miR-378*	2.29	0.4961
hsa-miR-141	2.89	0.3926	hsa-miR-380*	2.31	0.4251
hsa-miR-205	3.13	0.2706	hsa-miR-661	2.31	0.5051
hsa-miR-129-3p	3.53	0.1788	hsa-miR-10a	2.34	0.1452
hsa-miR-188-5p	3.58	0.5457	hsa-miR-148b*	2.34	0.4926
hsa-miR-107	3.81	0.1843	hsa-miR-592	2.35	0.4694
hsa-miR-183	3.91	0.1201	hsa-miR-449b	2.36	0.526
*hsa-miR-296-5p*	*4.26*	*0.084*	hsa-miR-487a	2.36	0.511
*hsa-miR-23a*	*4.58*	*0.1771*	hsa-miR-199a-3p	2.37	0.4496
*hsa-miR-22*	*4.78*	*0.3268*	hsa-miR-181a	2.41	0.3499
*hsa-miR-801*	*5.87*	*0.127*	hsa-miR-30a*	2.48	0.3854
*hsa-miR-200a*	*6.22*	*0.3678*	hsa-miR-144*	2.51	0.5976
*hsa-miR-203*	*6.22*	*0.1755*	hsa-miR-509-3p	2.56	0.4307
*hsa-miR-429*	*6.68*	*0.1588*	hsa-miR-146b-5p	2.57	0.224
hsa-miR-183*	*7.84*	*0.0203*	hsa-miR-154*	2.57	0.3571
hsa-miR-200b*	*8.02*	*0.1744*	hsa-miR-638	2.61	0.4688
*hsa-miR-601*	*8.31*	*0.2335*	hsa-miR-328	2.61	0.3059
*hsa-miR-200b*	*9.41*	*0.1332*	hsa-miR-656	2.62	0.4201
*hsa-miR-200c*	*9.77*	*0.1334*	hsa-miR-146b-3p	2.68	0.328
hsa-miR-200a*	*9.86*	*0.0974*	hsa-miR-202	2.77	0.3485
*hsa-miR-452*	*17.94*	*0.0238*	hsa-miR-548b-5p	2.77	0.4288
*hsa-miR-650*	*18.46*	*0.1454*	hsa-miR-340	2.78	0.2592
*hsa-miR-378*	*28.01*	*0.3958*	hsa-miR-15a*	2.81	0.2874
*hsa-miR-513-3p*	*229.63*	*0.355*	hsa-miR-29c*	2.87	0.4847
			hsa-miR-29a*	2.89	0.3125
			hsa-miR-132	2.91	0.2681
			hsa-miR-345	2.98	0.0946
			hsa-miR-214	3.02	0.4376
			hsa-miR-376a	3.05	0.4487
			hsa-miR-20a*	3.11	0.183
			hsa-miR-539	3.12	0.4698
			hsa-miR-487b	3.19	0.4356
			hsa-miR-433	3.21	0.3942
			hsa-miR-639	3.21	0.3717
			hsa-miR-885-5p	3.27	0.3696
			hsa-miR-520c-3p	3.28	0.3564
			hsa-miR-127-3p	3.29	0.4056
			hsa-miR-99a*	3.32	0.2645
			hsa-miR-361-5p	3.39	0.1345
			hsa-miR-134	3.41	0.369
			hsa-miR-130b*	3.51	0.3143
			hsa-miR-493	3.53	0.3315
			hsa-miR-212	3.58	0.241
			hsa-miR-19b-1*	3.68	0.3595
			hsa-miR-30d*	3.74	0.091
			hsa-miR-760	3.75	0.4218
			hsa-miR-125b-1*	3.77	0.3397
			hsa-miR-140-5p	3.81	0.1951
			hsa-miR-34b*	3.97	0.4022
			hsa-miR-99b*	*4.17*	*0.214*
			*hsa-miR-889*	*4.17*	*0.294*
			*hsa-miR-516a*	*4.17*	*0.244*
			*hsa-miR-770*	*4.17*	*0.274*
			*hsa-miR-671*	*4.55*	*0.174*
			*hsa-miR-100*	*4.76*	*0.204*
			*hsa-miR-543*	*4.76*	*0.264*
			*hsa-miR-410*	*5.00*	*0.285*
			*hsa-miR-495*	*5.00*	*0.385*
			*hsa-miR-485*	*5.26*	*0.325*
			*hsa-miR-411*	*5.56*	*0.235*
			hsa-miR-16-1*	*5.56*	*0.045*
			*hsa-miR-382*	*5.88*	*0.345*
			hsa-miR-100*	*5.88*	*0.225*
			hsa-miR-7-1*	*5.88*	*0.505*
			*hsa-miR-337*	*6.25*	*0.756*
			*hsa-miR-451*	*6.25*	*0.236*
			*hsa-miR-99a*	*7.14*	*0.167*
			*hsa-miR-218*	*7.69*	*0.847*
			*hsa-miR-370*	*7.69*	*0.327*
			*hsa-miR-379*	*8.33*	*0.788*
			*hsa-miR-409*	*9.09*	*0.369*
			*hsa-miR-376c*	*10.00*	*0.1110*
			*hsa-miR-432*	*11.11*	*0.2111*
			hsa-miR-214*	*12.50*	*0.1412*

All miRNA listed here presented a FDR-adjusted p value <0.3. The 42 miRNAs selected as differentially expressed are marked in italics.

Due to the scarcity of RNA quantity in many samples, it would be impossible to evaluate all 15 differentially expressed miRNAs selected in all samples. After a deep review of the available literature data from the 15 miRNAs identified in the discovery series, miR-452, miR-200c, miR-183* and miR-296-5p were selected for further analyses. The expression levels of these four selected miRNAs were determined in the entire cohort of samples (20 radioresistant and 14 radiosensitive) and, by using the median value as threshold cutoff, the miRNA expression was scored as low level, below the median value, and high level, above the median value (Additional file 2: Table S2).

### miR-296-5p expression is associated with patient prognosis

The expression levels (high or low) of miR-452, miR-200c, miR-183* and miR-296-5p were analyzed for potential correlations with clinical characteristics of the LSCC patients, including age, gender, tobacco consumption, tumor site, tumor stage, and radiosensitivity. The high expression of miR-296-5p showed a significant correlation with resistance to radiotherapy (p = 0.010; Table 3). No associations were observed between the clinical features and expression status of the other miRNAs tested.

**Table 3:** Correlation between the expression level of microRNAs miR-296-5p, miR-452, miR-183* and miR-200c and the clinical and pathological characteristics of the LSCC patients enrolled in the study

Variable	Categories	Number of cases	miR-296-5p		miR-452		miR-183*		miR-200c	
			Low	High	Low	High	Low	High	Low	High
			n (%)	n (%)	n (%)	n (%)	n (%)	n (%)	n (%)	n (%)
Age (years)	≤60 years	16	6 (35.3)	10 (58.8)	8 (47.1)	8 (47.1)	8 (47.1)	8 (47.1)	8 (50.0)	8 (47.1)
	>60 years	18	11 (64.7)	7 (41.2)	9 (52.9)	9 (52.9)	9 (52.9)	9 (52.9)	8 (50.0)	9 (52.9)
	*p* (2-sided)		NS		NS		NS		NS	
Gender	Female	4	2 (11.8)	2 (11.8)	2 (11.8)	2 (11.8)	2 (11.8)	2 (11.8)	2 (12.5)	2 (12.5)
	Male	30	15 (88.2)	15 (88.2)	15 (88.2)	15 (88.2)	15 (88.2)	15 (88.2)	14 (87.5)	15 (88.2)
	*p* (2-sided)		NS		NS		NS		NS	
Smoking	No	3	0 (0)	3 (17.6)	1 (5.9)	2 (11.8)	0 (0)	3 (17.6)	2 (12.5)	1 (9.1)
	Yes	31	17 (100)	14 (82.4)	16 (94.1)	15 (88.2)	17 (100)	14 (82.4)	14 (87.5)	16 (94.1)
	*p* (2-sided)		NS		NS		NS		NS	
Tumor site*	Glottic	27	15 (88.2)	12 (70.6)	14 (82.4)	13 (76.5)	14 (82.4)	13 (76.5)	14 (87.5)	12 (70.6)
	Supraglottic	7	2 (11.8)	5 (29.4)	3 1 (7.6)	4 (23.5)	3 (17.6)	4 (23.5)	2 (12.5)	5 (29.4)
	*p* (2-sided)		NS		NS		NS		NS	
Tumor stage	I	16	7 (41.2)	9 (52.9)	9 (52.9)	7 (41.2)	7 (41.2)	9 (52.9)	7 (43.8)	9 (52.9)
	II	18	10 (58.8)	8 (47.1)	8 (47.1)	10 (58.8)	10 (58.8)	8 (47.1)	9 (56.2)	8 (47.1)
	*p* (2-sided)		NS		NS		NS		NS	
Radiosensibility	Sensivite	14	11 (64.7)	3 (17.6)	8 (47.1)	6 (35.3)	9 (52.9)	5 (29.4)	7 (43.8)	7 (41.2)
	Resistant	20	6 (35.3)	14 (82.4)	9 (52.9)	11 (64.7)	8 (47.1)	12 (70.6)	9 (56.2)	10 (58.8)
	*p* (2-sided)		*0.01*		NS		NS		NS	

Italic value indicates statistical significance at *p* < 0.05.

*p* (*Fisher’s exact*); NS (p > 0.05).

**Fig. 1:**
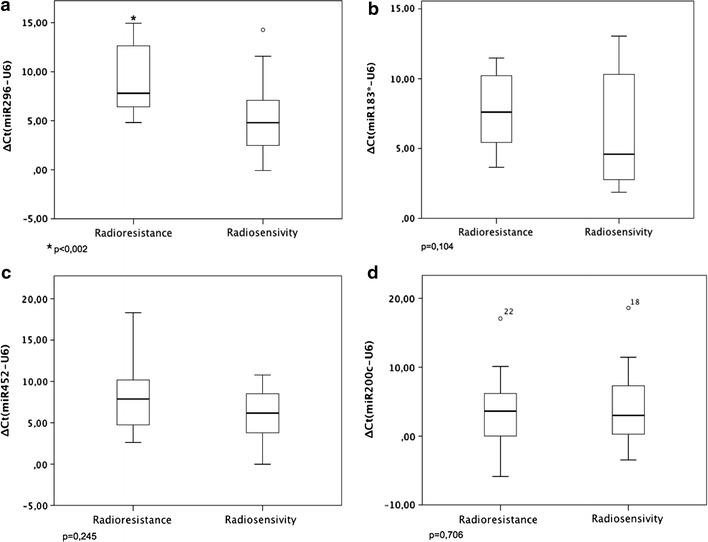
Comparison between radioresistant and radiosensitive groups relative to expression level (Mann–Whitney test): **a** miR-296-5p, **b** miR-183, **c** miR-452, **d** miR-200c.

The comparison of the expression of miR-296-5p between the radioresistant and radiosensitive groups reveals a significant higher expression of this miRNA in the radioresistant group (p = 0.002, Fig. 1). Expression of miR-452, miR-200c and miR-183* between the radioresistant and radiosensitive groups were not significant associated with radioresistance (Fig. 1).

**Fig. 2:**
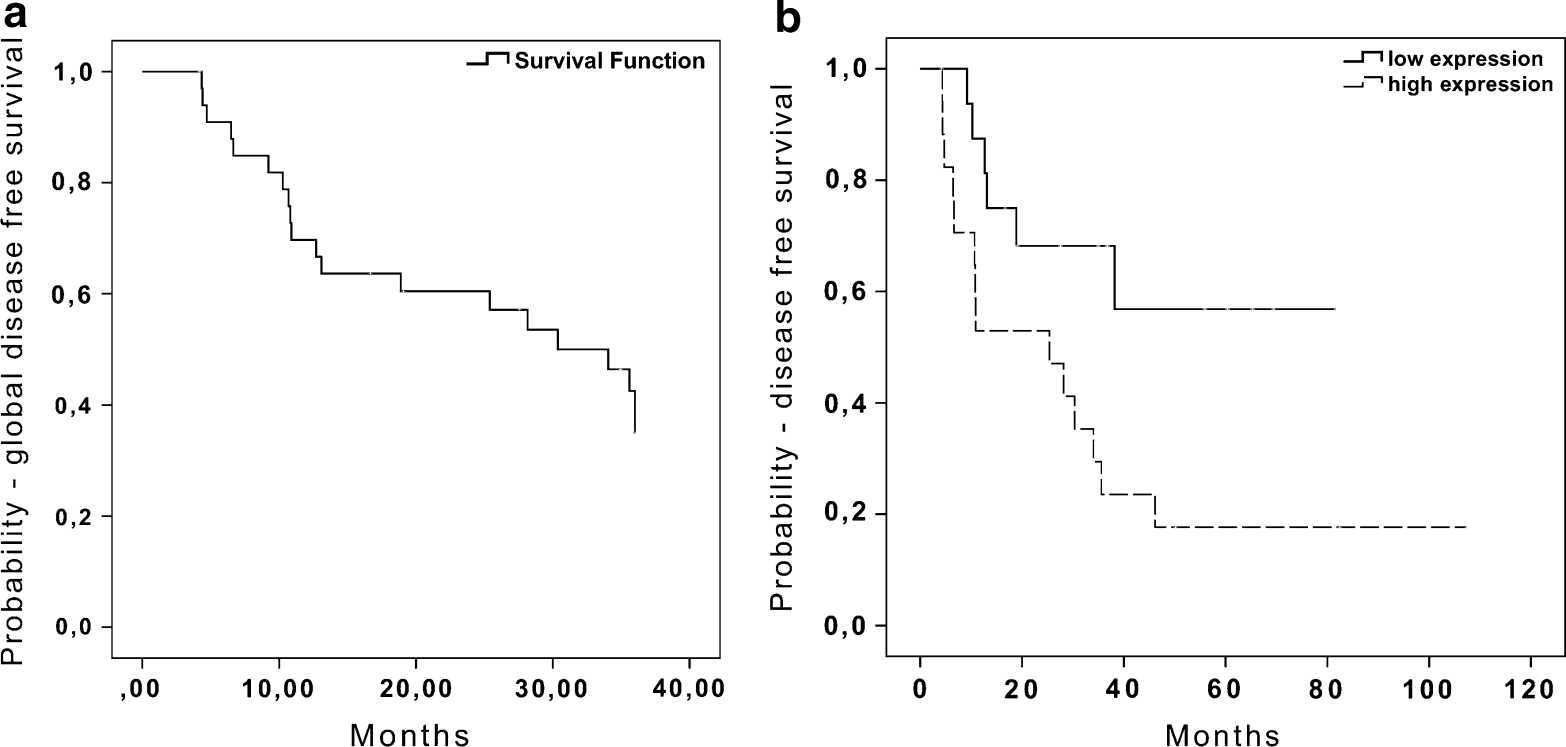
Survival curves for 34 laryngeal squamous cell carcinoma patients. **a** 3-years disease-free survival for all patients. **b** 3-years disease-free survival rate according to miR-296-5p expression level in LSCC samples.

The 3-year disease-free survival (DFS) rate considering all patients included in the study was 34% (Fig. 2a). Additionally, we investigated the association between the expression levels of the four selected miRNAs and the 3-year DFS. According to this analysis, 41.6% of the patients with low expression of miR-296-5p presented recurrences, while relapses were detected in 82.4% of the LSCC patients with high expression of this miRNA, and this difference was statistically significant (p = 0.025, OR = 8.6, 95 CI 1.7–42.2, Fig. 2b). Expression levels of miR-452, miR-200c and miR-183* were not associated with disease-free survival in this cohort (data not shown).

## Discussion

Despite recent advances in the multidisciplinary management of early stage laryngeal cancer, including surgical resection or larynx-preservation protocols, a substantial proportion of patients with localized or locally advanced disease will eventually relapse and die [25]. Radiotherapy as the initial treatment is commonly used, offering a possibility to preserve laryngeal function and reserving surgery as a salvage procedure. Nodal involvement, gender, tumor volume, impaired vocal cord mobility, tumor invasion of cartilage, and overall treatment time are factors found to be of prognostic importance. However, these prognostic factors fail to differentiate patients with good outcome of radiotherapy from those patients that will not respond to the treatment. In spite of the high rates of response to radiotherapy, around 30% of patients with small, good prognosis tumors are not cured by this treatment and must undergo salvage laryngectomy (partial or total). Therefore, an accurate and reproducible estimation of prognosis in patients with early laryngeal cancer is important as an opportunity to spare patients from ineffective and toxic therapies.

To better understand the biology of radiation response, analysis of the phenotype of tumor cells has been underway for the last several years. Previous studies have evaluated the prognostic significance of various biomarkers including angiogenesis markers, members of the pro-apoptotic family, cell cycle regulators, and proliferation markers in head and neck cancer [19, 20, 26–28]. However, results have been quite mixed, reflecting the multitude of factors that contribute to the complex tumor biology as well as the heterogeneity of laryngeal cancers in terms of biology, site, stage, and prognosis [20].

Several studies have reported specific miRNA expression in different cancer cell types and in distinct differentiation tumor stages [21, 29]. Some of them have exploited the potential of miRNAs as prognostic markers in HNSCC and found associations between the low levels of miRNAs such as miR-205 and let-7d and high levels of miR-451 with disease progression and increased risk for local and regional recurrence [30]. As diagnostic markers, the ratio between the expression of miR-221 and miR-375 was able to discriminate HNSCC and normal samples with a specificity of 93% and a sensitivity of 92% [31]. Moreover, although a small number of cases was evaluated, Fletcher et al. found a specific expression of miR-205 in metastatic lymph nodes of HNSCC patients [32].

Specifically in laryngeal cancer, five miRNAs (miR-331-3p, miR 603, miR, 1303, miR-660-5p and miR-212-3p) were identified specifically in plasma samples from affected patients, while theis expression was absent in the normal population. Therefore, these findings suggest miRNAs as promissor biomarkers for laryngeal cancer early diagnostic [33]. Cao et al. identified that miR-21, miR-93, miR-205, and miR-708 were upregulated and miR-125b and miR-145 were downregulated between adjacent normal tissue and laryngeal cancer, these results points to the importance of miRNAs in tumorigenesis and tumor progression of laryngeal cancer [34].

In turn, some studies have exploited the potential role of miRNAs in modulating radiosensitivity in different tumors. Given the fact that the local control of early-stage laryngeal cancers fails in one third of the patients treated with definitive radiotherapy, we sought to identify miRNA-based markers which expression profile could discriminate between radiosensitive patients, who could actually benefit from this treatment approach, and radioresistant ones.

Cancer stem cells are known to play an important roll in radiation resistance and this important biological characteristic may be mediated through miRNA regulation [35, 36]. Huang et al. conducted a study in which laryngeal cancer stem cells were successfully isolated and than irradiated. The miRNA expression analyzes were performed before and after radiation and 70 miRNA were found to be differentially expressed. This experiment in cell lines demonstrated that laryngeal stem cells have different behavior in response to radiation, so tumor radioresistance observed in clinical practice may be intermediated by laryngeal stem cells through miRNA disorders [37]. The better understanding of miRNAs is essential for early interventions and better clinical results.

Of note, other studies have indicated miRNAs as important mediators of radioresistance in human tumors without focusing in stem cells, such as non-Hodgkin’s lymphoma, oral squamous cell carcinoma, esophageal and lung cancers. Wu et al. (2012) showed that miR-148b is able to enhance radiosensitivity of non-Hodgkin’s lymphoma cells by promoting radiation-induced apoptosis [38]. As such, the involvement of miRNAs in the radiation resistance of esophageal cancer cell was established by Su et al. [39]. Shiiba et al. demonstrated that down-regulated miR-125b expression was associated with radioresistance mechanisms in oral squamous cell carcinoma and they suggested that the control of the expression or activity of miR-125b might contribute to overcoming radioresistance in this oral malignancy [40]. Oh et al. found overexpression of miR-let-7a in radiosensitive A549 lung cancer cells in a miRNA profile study [41], while Grosso et al. showed that lung cancer cells expressing miR-210 exhibit a radioresistance similar to that found in hypoxic control cells [42]. According to this study, miR-210 stable expression mimics hypoxia-induced metabolic changes associated with a slight but significant stabilization of HIF-1α, and this information, combined with a strong reduction of radioresistance following HIF-1 silencing, reinforces the central role of HIF-1 in the resistance to radiotherapy. The molecular mechanism responsible for this radioresistance is not fully understood and is likely mediated by a complex network of miR-210 targets involved in a wide set of biological functions, including cell cycle control, survival, DNA repair and cell metabolism.

Our study was the first to evaluate the miRNA expression profile of LSCC and found miR-296-5p up-regulated in radioresistant patients and this high expression significantly correlates with tumor recurrence. The microRNA miR-296-5p is involved in many physiological and pathologic processes, such as angiogenesis, insulin metabolism, tumorigenesis and fetal alcohol syndrome [43–45]. Using a miRNA microarray approach to analyze the miRNA expression profile in esophageal cancer, Hong et al. showed that miR-296-5p might mediate drug resistance at least in part through regulation of MDR1 and apoptosis [46]. Furthermore, these authors also demonstrated that esophageal tumors with high expression of miR-296-5p had a worse prognosis compared with those with low expression. Along the same line, our findings also correlated the high expression of miR-296-5p with worse recurrence-free survival for early-stage LSCC patients.

The possibility of evaluating small fragments from early laryngeal carcinoma biopsies to predict the response to definitive radiotherapy is relevant given the difficulty of differentiating non-responder patients at high risk of relapse that should receive a more aggressive treatment, obviating potentially hazardous overtreatment in the group with a presumed favorable outcome. Based in the results present here, we may speculate that the expression of miR-296-5p in biopsy of laryngeal cancers could be a helpful biomarker to identify subjects resistant to radiotherapy. It is worth mentioning that the present study has some limitations. Remarkably, the patient number was limited. Therefore, further validation of these results requires studies with larger patient groups, which will allow the determination of cutoff values to easily classify the miR-296-5p expression level as “high” or “low”. So, only after the establishment of these cutoffs and by achieving a good predictive negative value, this molecular approach could constitute a valuable tool to predict the radiotherapy response in a biopsy of the primary tumor, helping the clinicians in the adoption of the most effective treatment for early LSCC patients.

## Conclusions

In the present study, the only one to our knowledge exploring the prognostic role of miRNAs in early laryngeal cancer in radiosensitive and radioresistant patients, we have shown that high expression level of miR-296-5p is an adverse prognostic factor for recurrence in patients with early stage LSCC treated with definitive radiotherapy. Moreover, the evaluation of the miR-296-5p expression could help clinicians to discriminate among LSCC patients who will take benefit from radiotherapy and who should take surgery as the first curative attempt.

## Additional files

Additional file 1:
**Table S1.** Raw data of the expression level of 667 human microRNAs in LSCC samples according to the TaqMan Human MicroRNA Array assays. Additional file 2:
**Table S2.** Expression data of the four selected microRNAs in the 34 LSCC samples included in the study. Ct values represent the average of three technical replicates.
